# Role of γ-Aminobutyric Acid (GABA) as an Inhibitory Neurotransmitter in Diabetes Management: Mechanisms and Therapeutic Implications

**DOI:** 10.3390/biom15030399

**Published:** 2025-03-11

**Authors:** Hassan Barakat, Thamer Aljutaily

**Affiliations:** Department of Food Science and Human Nutrition, College of Agriculture and Food, Qassim University, Buraydah 51452, Saudi Arabia; thamer.aljutaily@qu.edu.sa

**Keywords:** GABA, diabetes mellitus, immunomodulation, mechanisms, food supply

## Abstract

GABA (γ-Aminobutyric Acid), a well-established inhibitory neurotransmitter in the central nervous system, has garnered considerable interest for its potential role in diabetes management, particularly due to its presence in pancreatic islets. This review aims to explore the therapeutic role of GABA in diabetes management and its potential mechanisms for antidiabetic effects. Relevant studies were searched across databases such as PubMed and ScienceDirect, applying strict eligibility criteria focused on GABA administration methods and diabetic models. The collective results showed that the administration of GABA in diabetic models resulted in remarkable enhancements in glucose and insulin homeostasis, favorable modifications in lipid profiles, and amelioration of dysfunctions across neural, hepatic, renal, and cardiac systems. The findings from the literature demonstrated that GABAergic signaling within pancreatic tissues can significantly contribute to the stimulation of β cell proliferation through the facilitation of a sustained *trans*-differentiation process, wherein glucagon-secreting α cells are converted into insulin-secreting β-like cells. In addition, activated GABAergic signaling can trigger the initiation of the PI3K/AKT signaling pathway within pancreatic tissues, leading to improved insulin signaling and maintained glucose homeostasis. GABAergic signaling can further function within hepatic tissues, promoting inhibitory effects on the expression of genes related to gluconeogenesis and lipogenesis. Moreover, GABA may enhance gut microbiota diversity by attenuating gut inflammation, attributable to its anti-inflammatory and immunomodulatory properties. Furthermore, the neuroprotective effects of GABA play a significant role in ameliorating neural disorders associated with diabetes by facilitating a substantial reduction in neuronal apoptosis. In conclusion, GABA emerges as a promising candidate for an antidiabetic agent; however, further research is highly encouraged to develop a rigorously designed framework that comprehensively identifies and optimizes the appropriate dosages and intervention methods for effectively managing and combating diabetes.

## 1. Introduction

For over 70 years, γ-aminobutyric acid (GABA) has been recognized primarily as an inhibitory neurotransmitter predominantly found in brain tissues [1]. In the early 1970s, research identified its presence in substantial quantities within pancreatic tissues, indicating a broader physiological role than previously understood [2]. The concentrations detected in these tissues may reach approximately 50% of the quantities identified in cerebral tissues [3,4]. This discovery has prompted further investigation into the physiological existence of GABA beyond its traditional context within the nervous system, particularly concerning pancreatic islets. Recent research has shown that pancreatic islet cells, particularly β cells, have the capacity to biosynthesize GABA in a manner similar to that observed in neural tissues [5]. Research has also identified the presence of GABA receptors on the membranes of different pancreatic islet cells, including both α and β cells, indicating that active GABAergic signaling may occur within the pancreas [6]. Furthermore, the distribution pattern of GABA in pancreatic tissue was found to closely resemble that of insulin [7]. This observation suggests a possible interaction between GABA and the secretion or functionality of insulin, underscoring the need for further investigation into their relationship. Given that insulin constitutes a pivotal element in regulating glucose homeostasis [8], thereby understanding the mechanisms by which GABA influences its secretion may produce significant implications for the management and therapeutic approaches related to diabetes.

Diabetes is a multifaceted metabolic disorder characterized by chronic hyperglycaemia, which significantly impacts individuals’ health and overall wellness. Consequently, there has been considerable attention and investment in research aimed at developing and implementing strategies for diabetes management over the past decade. Understanding the fundamental mechanisms that contribute to the progression of diabetes and identifying specific factors associated with the condition are of critical importance in order to establish the formulation of more effective therapeutic strategies [9,10,11]. In this context, research has indeed demonstrated a notable reduction in GABA concentrations in serum samples from individuals diagnosed with type 2 diabetes (T2D) and those exhibiting impaired glucose tolerance [12]. Furthermore, a comparative analysis of pancreatic islets derived from T2D donors revealed a substantial decrease in overall GABA concentration compared to islets obtained from nondiabetic donors. Specifically, the quantification of GABA in islets harvested from T2D donors yielded mean measurements of 1.6 nmol/mg protein, whereas islets from nondiabetic donors recorded mean measurements of 7.7 nmol/mg protein [6].

Most recent T1D management research has been reviewed by Mick and McCormick [13]. They indicated that GABA is a safe and inexpensive therapeutic agent for T1D, with a unique physiological role in pancreatic islets and the gut. It has demonstrated potential in increasing insulin secretion, suppressing glucagon release, and dampening T-cell-mediated autoimmune processes. Clinical studies indicate that oral GABA is well-tolerated, although higher doses may be necessary for significant effects. GABA’s mechanisms include autocrine and paracrine effects on islet cells, promoting b-cell survival and proliferation while dampening autoimmune responses. The research suggests that GABA-producing microbiota may play a protective role against T1D through immunomodulation. The study faced challenges related to the low dose of GABA, which may have limited its effectiveness in achieving desired outcomes. Medication adherence was another significant challenge, as compliance was measured at an average of 83%, with some visits recording only 50% adherence. The results of immunophenotyping isolated peripheral blood mononuclear cells did not ideally mimic the localized immune response within the pancreatic islet, indicating a potential gap in understanding the immune dynamics. Additionally, the short half-life of oral GABA may have contributed to insufficient islet GABA exposure to elicit an antidiabetic effect. Therefore, future research should explore combination therapies and the role of GABA-producing microbiota in T1D prevention and treatment.

Khachatryan et al. [14] illustrated that the GABA-supporting mixture (GSM) exhibits a significant hypoglycemic effect in a streptozotocin-induced diabetes model in rats. GSM is capable of restoring the activity of glutaminase (GLS) and glutamate decarboxylase (GAD), which are reduced in diabetic animals. Increased levels of GABA, facilitated by GSM, are hypothesized to be beneficial in diabetes treatment, suggesting that GSM may serve as an additional therapeutic approach for diabetes management. Further research is needed for long-term effects and optimal component ratios. Additionally, gamma-aminobutyric acid (GABA) significantly influences the antioxidant capacity by regulating the synthesis of various compounds and enhancing antioxidant enzyme activity. The mechanisms through which GABA controls ROS are vital for plant resilience against stress [15].

These insights and findings strongly highlight the potential involvement of GABA in the pathophysiological mechanisms underlying diabetes. Therefore, this review seeks to evaluate the existing literature investigating the potential association between GABA and the progression of diabetes. This aims to clarify the underlying mechanisms functioning within the pancreatic system while also considering the related roles within the nervous system.

## 2. Search and Data Collection Strategy

A thorough investigation was carried out utilizing multiple databases such as PubMed and ScienceDirect and additional searches through Google Scholar to ensure a comprehensive literature review. The following keywords were incorporated: “GABA”, “γ-Aminobutyric Acid”, and “Diabetes”. The primary aim of this research was to critically review and synthesize relevant studies and scholarly articles that investigate the relationship between GABA and diabetes management. The search was directed toward studies that evaluate GABA’s effects in various experimental contexts, including animal models, in vitro studies, and human clinical trials. To qualify for inclusion in this review, studies must meet the following eligibility criteria: (i) administration of GABA via direct oral consumption, injection, or dietary enrichment/supplementation; and (ii) utilization of diabetic models, which could include diabetic patients, induced-diabetic animal models, or diabetes-related tissues. Eligible studies should provide significant outcomes of key diabetic-related parameters, such as blood glucose levels, lipid profiles, insulin signaling pathways, or the functionality of vital organs. A total of 18 eligible studies were included in the review. Among these, two human clinical trials were conducted in Sweden and the United States. The remaining studies comprised experimental research utilizing animal models, along with one study focused on cell lines.

In addition, there was an emphasis on studies that investigate and examine the possible mechanisms of action involved. To explore the potential for a comprehensive understanding of GABA’s role in influencing glucose metabolism, insulin sensitivity, and other relevant factors in individuals with diabetes. The data collection was completed by 15 October 2024, ensuring that all relevant information was gathered promptly.

## 3. Overview and Biosynthesis

GABA is a four-carbon nonprotein-free amino acid widely distributed in prokaryotic and eukaryotic organisms. It has been identified in all parts of every studied plant, with initial detection occurring in potato tubers. Later research explored the occurrence of GABA in animals, where its presence was first observed in the brain. The essential role of GABA as a neurotransmitter within the central nervous system of mammals subsequently became evident. GABA exhibits a remarkable concentration within neuronal cells, with levels approximating 1000 times greater than those of any other neurotransmitter in the same region [16].

### 3.1. GABA in the Nervous System

The biosynthesis of GABA is initiated by the amino acid glutamate, catalyzed by the enzyme glutamate decarboxylase (GAD), with the presence of vitamin B6 (pyridoxine) as a cofactor. This biosynthetic process occurs within the cytoplasm of presynaptic neurons. Once synthesized, GABA is loaded into synaptic vesicles via the vesicular inhibitory amino acid transporter. It is then released in response to nerve signals, allowing it to bind to receptors or be reabsorbed for degradation. GABA can interact with two main types of receptors: GABA_A_ receptors and GABA_B_ receptors. Both receptor types enable GABA to function as an inhibitor, preventing excessive signaling between neuronal cells [17]. GABA plays a pivotal role in maintaining balanced cerebral functions by modulating neural excitability. This modulation is critical in the regulation of a range of neurological conditions, including sleep disorders, anxiety disorders, mood dysregulation, depression, epilepsy, schizophrenia [18], and autism spectrum disorder [19].

### 3.2. GABA in the Endocrine System

The presence of GABA outside the central nervous system was initially detected in the early 1950s. Its presence was confirmed in various endocrinal tissues, including the pancreas, adrenal glands, kidneys, gastrointestinal tract, and placenta. However, the concentrations detected in all of these tissues were sufficiently low to imply that the brain serves as the principal organ responsible for sustaining significant levels of GABA [1]. Later in the 1970s, researchers discovered a quantifiable existence of GABA in the pancreatic islets [2]. Subsequent studies have shown that GABA is found in the pancreatic tissues at concentrations approximately half of those found in brain tissues (approximately 20 mmol/g vs. 40 mmol/g, respectively). In contrast, GABA levels in other tissues are typically below 1 mmol/g [4,20]. A study even found that GABA concentration within the islets is comparable to those found in neurons [3].

Further investigations have demonstrated that GAD is also present in considerable concentrations within the pancreatic islets. This indicates that GABA production can occur within pancreatic tissues, similar to its synthesis observed in the nervous system. In particular, the expression of GAD was predominantly localized in the β cells, indicating that these cells are the primary site for the endocrine synthesis of GABA [5]. Upon the catalytic conversion of GAD into GABA, the resultant GABA molecules are subsequently stored in synaptic-like microvesicles located within the pancreatic β cells, specifically within the insulin granules. The released GABA molecules are then capable of functioning through the interaction with GABA receptors found in the pancreatic tissues [6].

The primary type of receptors identified in human pancreatic islets are the GABA_A_ receptors, in contrast to the presence of both GABA_A_ and GABA_B_ receptor types observed in cerebral tissues. The expression of GABA_A_ receptors has been observed in insulin-secreting β cells, glucagon-secreting α cells, and somatostatin-secreting δ cells [6]. This expression pattern highlights a unique aspect of GABA signaling in human islets compared to other species, such as rats and guinea pigs, where functional GABA_A_ receptors are absent in their β cells [21,22]. It was identified by Korol et al. [6] that there are two distinct subtypes of GABA_A_ receptors present in human β cells: islet-GABA_A_ receptor I (iGABA_A_RI) and islet-GABA_A_ receptor II (iGABA_A_RII). These GABA_A_ receptors serve as biological sensors for GABA. Once activated, they attenuate the rate of exocytosis, consequently modulating glucose-stimulated insulin secretion. This highlights the critical role of GABA signaling in regulating insulin release.

## 4. Role of GABA in Diabetes Management

Recent investigations have provided substantial evidence regarding the potential of GABA as a therapeutic agent for diabetes management, as summarized in Table 1.

The data presented indicate that the administration of GABA exhibits promising effects in improving glucose, lipid, and insulin homeostasis under diabetic conditions. In addition, the findings reveal beneficial outcomes in mitigating organ dysfunctions associated with diabetes. This section seeks to elucidate the potential mechanisms through which GABA mediates these advantageous effects.

### 4.1. Neurological Protection

Considering the fact that the central nervous system is recognized as the main site where GABA operates and exerts its effects, decreased GABA concentrations under diabetic conditions may indeed be linked to or associated with the emergence and development of various neurological complications that are often observed in patients suffering from diabetes [12,41]. In the parietal lobe and hippocampus of db/db mice, a model resembling T2D, the GABA concentrations were significantly lower than those in normal mice [42]. Research has found that GABAergic neurons, a specific type of neurons that primarily employ GABA as their neurotransmitter, exhibit increased susceptibility to hyperglycemia, consequently leading to their dysfunction. GABAergic neurons play a crucial role in promoting proper brain function and cognitive processes by inhibiting the activity of other neurons, thus maintaining a balance between excitation and inhibition in the brain. Therefore, their dysfunction can significantly impact neural activities in individuals diagnosed with diabetes, contributing to conditions such as encephalopathy [43]. Hyperglycemia can disrupt GABAergic signaling by slowing down the response time of GABA receptors within neuronal structures. This implies that the engagement of GABA with its receptors, in addition to the subsequent signaling processes, may experience delays or modifications leading to the progression of neuropathological conditions associated with diabetes [44]. Research has also demonstrated that alterations in GABAergic signaling activity can similarly occur as a consequence of hypoglycemia. A notable decline in the presence of GABA receptors has been reported in brain tissues of both hypoglycemic and diabetic rodent models. Moreover, both models exhibited a reduction in the glutamate decarboxylase (GAD) enzyme activity, which is responsible for the catalysis of GABA synthesis [45].

**Figure 1:**
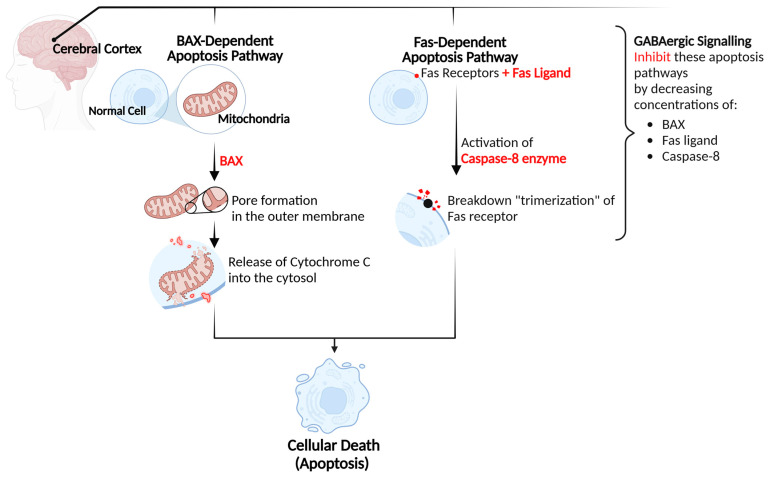
GABA’s mechanism of action in promoting neuroprotective effects.

Conversely, interventions involving GABA in diabetic models have demonstrated encouraging outcomes in mitigating the complications associated with diabetes, including but not limited to neurological functions (Table 1). In diabetic rats administered with GABA tea extract, the results significantly showed a reduction in autophagy within the cerebral cortex. The findings showed that GABA can promote significant protective effects against diabetic-induced neuronal damage by inhibiting cellular apoptosis within the cerebral cortex. The findings were based on substantial reductions in the concentrations of Fas ligand and caspase-8 following the administration of GABA. Fas ligand is a protein that binds to Fas receptors located on cellular membranes, whereas caspase-8 is an enzyme that becomes activated upon this binding. This interaction leads to the trimerization or degradation of the Fas receptor, thereby initiating a series of molecular events known as the “Fas pathway”. Ultimately, these processes result in cellular apoptosis. Moreover, reductions in Bax, cytochrome c, and caspase-9 concentrations were also observed in the cerebral cortex of diabetic rats following GABA administration. Bax is a proapoptotic protein that facilitates cellular apoptosis through the formation of pores within the outer mitochondrial membrane, thereby allowing the release of proteins such as cytochrome c into the cytosol. The release of cytochrome c consequently leads to the activation of caspase-9, an enzyme that plays a significant role in the process of apoptosis and cellular signaling. Therefore, alleviations in the concentrations of Fas ligand, Bax, cytochrome c, and caspases indicate a reduction in cellular apoptosis dependent on both the Fas and mitochondrial pathways, resulting in neuroprotective effects [26], Figure 1.

### 4.2. Promotion of Pancreatic β Cell Proliferation

Research has demonstrated that the concentrations of GABA within the pancreatic islets exhibit a notable reduction as a result of diabetes onset [6]. In a study conducted in the early 2000s [7], researchers examined diabetic rat models. They found a significant decline of approximately 68% in GABA-like immunoreactive cells within the pancreatic islets compared to those in normal rats. These cells, characterized by the presence of GABA or compounds that exhibit reactivity to GABA, are crucial for understanding the role of GABA in the pancreas’s endocrine activities. Recent research has provided considerable evidence of GABA’s role in enhancing glucose metabolism and improving insulin sensitivity in various diabetic models [28,30,32,33,36,37]. Enhancements in the proliferation of insulin-secreting β cells have been extensively observed in diabetic models as a result of GABA interventions [27,28,33,34,38]. Interestingly, the distributional pattern of GABA within the pancreatic tissue closely resembles that of insulin [7], suggesting the correlation with increased insulin-secreting β cells observed following GABA treatments. GABA can significantly promote β cell proliferation by facilitating the transdifferentiation of α cells into β cells. This transdifferentiation phenomenon implies the conversion of glucagon-secreting α cells into insulin-secreting β-like cells, thereby restoring insulin homeostasis under diabetic conditions [34]. In pancreatic tissues of streptozotocin-induced mice, the administration of GABA significantly increased the size and number of islets, resulting in a substantial hyperplasia of β-like cells. It was shown that GABA has the ability to induce the conversion of α cells into β-like cells by downregulating the expression of Arx, a transcription factor that inhibits this conversion process. This downregulation triggers the reactivation of developmental pathways, leading to the compensatory formation of new α-cells. After prolonged GABA administration, these newly generated α-cells can be converted into β-like cells. This cycle of neogenesis and conversion ultimately results in the hyperplasia of β-like cells. Notably, the entire mass of β-cells exhibits the ability to regenerate at least twice following two cycles of streptozotocin-induced β-cell destruction in response to the administration of GABA. Human α-cells were shown to exhibit a similar capacity to convert into β-like cells upon maintained GABA exposure. The newly formed β-like cells exhibit functionality and possess the capability to significantly ameliorate the complications of chemically induced diabetes in animal models. Indicating that GABA not only facilitates the process of cellular conversion but also restores insulin synthesis [35].

**Figure 2:**
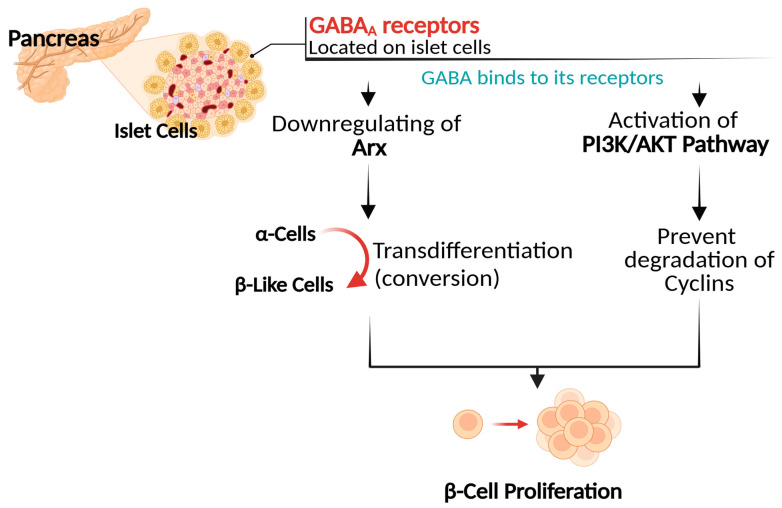
GABA’s mechanism of action in promoting β cell proliferation.

In comparison to alternative trans-differentiation approaches, such as islet transplantation and stem cell therapy, GABA-mediated trans-differentiation presents a potentially more accessible and less invasive alternative. Islet transplantation is a well-established treatment for diabetes, demonstrating proven clinical benefits. However, it is constrained by issues related to the scarcity of available donor islets and the requirement for immunosuppressive therapy [46]. Although the clinical efficacy of GABA-mediated trans-differentiation is still under investigation, it offers a promising avenue for future research in diabetes treatment. Stem cell therapy also presents a promising strategy for β-cell replacement and holds the potential for large-scale production of insulin-producing cells, however, its clinical application remains in its early stages. Notable challenges, including immune rejection and the risk of tumorigenicity, must be addressed [47,48]. In this context, GABA-mediated trans-differentiation may serve as a complementary approach to such approaches, enhancing the endogenous regeneration of β-cells and potentially reducing the reliance on exogenous cell transplantation, Figure 2.

Furthermore, studies have shown that GABA can promote the regeneration of β-cells by activating the phosphatidylinositol 3-kinase (PI3K)/Protein Kinase B (AKT) signaling pathway [33,39], a well-known cellular pathway acknowledged for its significance in the proliferation of β-cells due to its role in regulating physiological processes such as cellular growth, proliferation, survival, and metabolic functions [49,50]. GABA is capable of activating this signaling pathway through its interaction with GABA_A_ receptors located on islet cells [6]. This binding activates the PI3K enzyme, which in turn triggers the AKT protein. Once activated, AKT regulates specific pathways that prevent the degradation of cyclins, a group of proteins essential for regulating the cell cycle, thereby promoting cellular proliferation [49]. A recent study reported that the administration of GABA showed a significant increase in the levels of AKT within the hepatic tissue of mice subjected to a high-fat diet, such an increase was correlated with improved insulin resistance [33]. In addition, GABA’s role in activating the PI3K/AKT can further facilitate the upregulation of critical transcription factors involved in the development and regeneration of β cells, such as Pancreas/duodenum homeobox protein 1 (PDX1) and Neurogenin-3 (NEUROG3) [39]. PDX-1 is essential for maintaining β-cell identity and function, while NEUROG3 plays a role in the differentiation of endocrine cells in the pancreas. Overexpression of NEUROG3 was observed in type 1 diabetic models, suggesting a potential response to autoimmune inflammation and an effort at β cell regeneration [51].

As a further point of interest, a noteworthy finding indicated that the efficiency of GABAergic signaling within pancreatic tissues may depend on specific concentrations of GABA. Korol et al. reported [6] that pancreatic GABA_A_ receptors exhibit a pronounced sensitivity to GABA concentrations. This indicates that these receptors are remarkably efficient in catching GABA, even at minimal concentrations. They identified an optimal physiological concentration range of 100 to 1000 nM of GABA for activating GABA_A_ receptors within β cells. However, when GABA concentrations exceed this optimal range, the receptors tend to desensitize, resulting in diminished responsiveness and ultimately leading to a complete dysfunction. This heightened sensitivity underscores the critical necessity of sustaining GABA concentrations within a specific range to ensure proper receptor functionality and insulin secretion in pancreatic β cells. Nonetheless, further research is indeed recommended to develop a more comprehensive understanding of this critical point, Figure 2.

### 4.3. Gene Expression Regulation in Glucose and Lipid Metabolism

Increased availability of insulin as a result of improved β-cell mass, as discussed in an earlier section, can further lead to enhancements in the overall insulin signaling pathway. This enhancement subsequently results in improved glucose uptake and metabolic processes through the upregulation of genes involved in the insulin signaling pathway [52,53]. The intraperitoneal injection of GABA into type 2 diabetic rat models was found to increase the expression of insulin receptor substrate 1 (IRS1) and glucose transporter type 4 (GLUT4) [36,38]. IRS1 is a protein that facilitates the physiological response to insulin, whereas GLUT4 is a protein that facilitates the transport of glucose from the bloodstream into cells. The IRS1/GLUT4 pathway is considered as a target for therapeutic interventions in addressing metabolic disorders [54]. It is noteworthy that, in comparison to insulin therapy, GABA demonstrated superior efficacy in ameliorating glucose tolerance, reducing plasma glucose levels, and enhancing insulin sensitivity, attributable to its role in facilitating the expression of GLUT4 [38].

Furthermore, GABA is capable of functioning within hepatic tissues, given that the liver exhibits a high concentration of GABAergic signaling [33]. It has been found that GABA can promote alterations in the expression of gluconeogenesis-related genes within the liver [33,38]. The process of gluconeogenesis is a foundational concept within the metabolic system that implies the generation of glucose primarily in hepatocytes from noncarbohydrate carbon substrates, thereby maintaining glucose homeostasis. This process can be stimulated by different hormones and substrates, such as cortisol, epinephrine, and glucagon. The overexpression of gluconeogenesis is a significant contributor to hyperglycemia observed in diabetic individuals [55]. Reduction in the gene expression of glucagon receptors was observed in diabetic rats intraperitoneally injected with GABA. However, the findings did not reveal any significant alterations in the serum levels of glucagon [38]. Elevated glucagon levels are frequently observed in individuals with diabetes, particularly type 2 diabetes, contributing to hyperglycemia through gluconeogenesis stimulation [56]. This indicates that despite elevated glucagon levels, GABA exhibits a capacity to attenuate gluconeogenesis by reducing the liver’s sensitivity to glucagon via the down-regulation of glucagon receptors. Similar findings were observed in diabetic rat models fed with GABA-enriched wheat bran [31]; notable enhancements in glucose tolerance and insulin sensitivity were observed, attributed to GABA’s role in attenuating gluconeogenesis. The results showed a downregulation of two enzymes involved in the gluconeogenesis pathway reactions, phosphoenolpyruvate carboxykinase (Pepck), and glucose-6-phosphatase catalytic subunit (G6pc). This observation further supports the reduction in glucose synthesis, which contributes to the amelioration of glucose intolerance.

**Figure 3:**
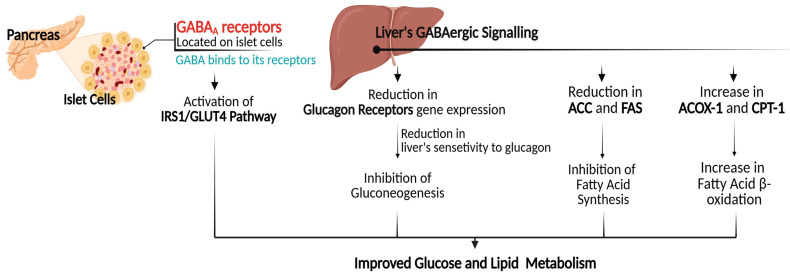
GABA’s mechanism of action in improving glucose and lipid metabolism. (IRS1): insulin receptor substrate 1; (GLUT4): glucose transporter type 4; (ACC): acetyl-CoA carboxylase; (FAS): fatty acid synthase; (ACOX-1): acyl-CoA oxidase 1; (CPT-1): carnitine palmitoyltransferase 1.

Hepatic GABAergic signaling pathways can further play roles in regulating the expression of genes related to lipogenesis, thereby contributing to reduced fat accumulation. The administration of GABA has been shown to inhibit the expression of key enzymes involved in hepatic lipid metabolism, particularly acetyl-CoA carboxylase (ACC), and fatty acid synthase (FAS) [33]. Both enzymes are pivotal in the pathogenesis of diabetes due to their regulatory effects on fatty acid synthesis. Elevated ACC/FAS activity leads to excessive fatty acid synthesis, which is associated with visceral fat accumulation, impaired insulin sensitivity, and increased circulating concentrations of fasting insulin [57]. GABA was further shown to promote the expression of genes related to fatty acid β-oxidation, including acyl-CoA oxidase 1 (ACOX-1), and carnitine palmitoyltransferase 1 (CPT-1). This upregulation in gene expression contributes to decreased hepatic fat accumulation with improved lipid profiles [33]. These findings underscore GABA’s critical role in lipid metabolism modulation, suggesting that targeting GABA pathways may present therapeutic potential for addressing metabolic challenges in individuals with diabetes, Figure 3.

### 4.4. Inflammation Relief and Immune Response Modulation

The hepatic GABAergic signaling pathways may further contribute to substantial attenuation of hepatic inflammation associated with diabetes [33]. In hepatic tissues of obese hyperinsulinemic rat models, the administration of GABA significantly resulted in decreased inflammatory cytokines, including interleukin-1 beta (IL-1β) and tumor necrosis factor-alpha (TNF-α) [33]. These cytokines are key mediators of inflammatory responses and are frequently elevated in conditions of insulin resistance, attributable to their impact on impairing insulin signaling pathways, leading to hepatic insulin resistance [58]. The capacity of GABA to alleviate hepatic inflammation was found to relate to its function in facilitating a substantial decrease in macrophage infiltration within the liver [33]. Macrophages are types of immune cells that infiltrate into the body’s tissues as a response to different stimuli, including infection, injury, or inflammation. However, excessive macrophage infiltration can exacerbate inflammation and induce tissue damage, thereby indirectly exacerbating insulin resistance [59,60]. These findings suggest a potential therapeutic role for GABA in treating complications associated with diabetes and obesity-related liver issues. Moreover, a reduction in the concentrations of TNF-α was likewise observed in the cardiac tissues of diabetic rat models following the administration of GABA tea [25]. It was found that GABA can alleviate cardiac inflammation by suppressing cardiomyocyte apoptosis through its ability to reduce the Fas-dependent apoptosis pathway in cardiac tissues. Consistent findings were likewise observed in diabetic rats administered with fermented-rich GABA Juice [29]; GABA’s intervention markedly exhibited anti-inflammatory properties, as evidenced by significant reductions in the serum levels of different inflammatory cytokines, including interferon–gamma (IFN–γ), IL-6, and transforming growth factor beta-1 (TGF-β1), all of which are associated with the progression of diabetes-related complications. Elevated levels of IFN-γ have been observed in diabetic conditions and are recognized as a contributing factor in the pathogenesis of type 1 diabetes, as it facilitates the degradation of β-cells [61,62]. Similarly, elevated levels of IL-6 are noted in instances of obesity and type 2 diabetes and are linked to chronic inflammation that ultimately results in the disruption of insulin signaling, thereby exacerbating insulin resistance [63,64]. Elevated levels of TGF-β1, on the other hand, have been shown to play a role in the development of diabetic nephropathy [65]. GABA was further found to improve the secretion of IL-10, an anti-inflammatory cytokine that plays a crucial role in modulating the immune response by mitigating excessive inflammatory processes [29]. A recent systematic review has found that a deficiency of IL-10 is commonly prevalent among diabetic individuals [66].

**Figure 4:**
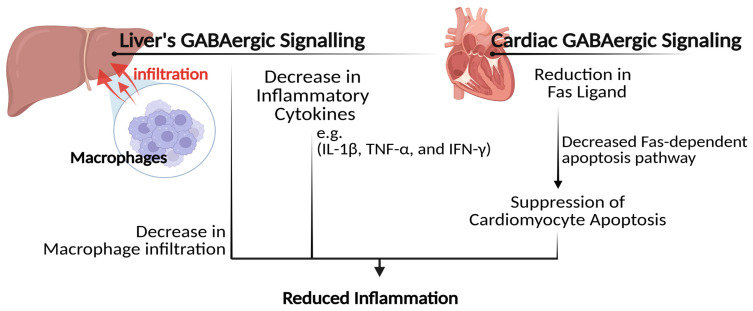
GABA’s mechanism of action in reducing inflammation. (IL-1β): interleukin-1 beta; (TNF-α): tumor necrosis factor-alpha; (IFN-γ): interferon–gamma.

Further investigation has revealed that GABA can regulate the secretion of a considerably greater number of cytokines than was previously acknowledged [67]. An in vitro study utilizing peripheral blood mononuclear cells isolated from individuals with type 1 diabetes demonstrated that exposure to GABA significantly inhibited the secretion of 47 distinct cytokines, including TNF-α, IL-13, IFN-γ, and TNF-β. GABA was also found to modulate cytokine secretion at higher concentrations further, indicating a concentration-dependent influence. These results were found to relate to GABA’s regulatory effects on the immune system. A pronounced suppressive effect on the proliferation of immune cells, particularly T helper cells, was observed following exposure to GABA, with a documented decrease of approximately 40%, Figure 4.

### 4.5. Improvements in Gut Microbiota

Recent research has demonstrated that changes in both circulating and cerebral concentrations of GABA are linked to alterations in gut microbiota composition [68]. Altered intestinal microbiota composition has been well-established as a significant factor in the pathogenesis of metabolic disorders, including diabetes [69,70,71]. For instance, gut microbiota produces short-chain fatty acids (SCFAs) as a byproduct of their metabolic activities. SCFAs have been shown to enhance insulin sensitivity by alleviating gut inflammation, a factor known to contribute to insulin resistance and the development of diabetes. Furthermore, SCFAs stimulate the secretion of hormones such as glucagon-like peptide-1 (GLP-1). This hormone is pivotal not only in promoting insulin secretion but also in regulating appetite effectively [70]. Individuals with T2D frequently exhibit unique gut microbial compositions compared to healthy individuals [69,71,72]. Zhang et al. [32] showed that administering GABA-rich sprouted beans to diabetic-induced models significantly improved gut microbial diversity within caecum tissues. These results were based on a notable reduction in the *Firmicutes*/*Bacteroidetes* ratio, which is a crucial parameter in microbiome research reflecting the relative abundance of two dominant bacterial phyla in the human gut. The results further showed an increase in the Chao index, a quantification of the overall diversity of microbial species. Zhang and his colleagues showed a notable abundance of beneficial bacteria such as *Lachnospiraceae*, *Verrucomicrobia*, and *Akkermansia*. Similar outcomes were observed in diabetic rats that were administered a low dosage of GABA supplementation [73]. A notable reduction in microbial diversity was observed exclusively in the nontreated rats, indicating the beneficial effects of GABA in improving gut health.

GABA may facilitate enhancements in gut microbiota composition by promoting a more favorable environment for the growth and abundance of beneficial bacteria within the gastrointestinal tract [68]. In enterotoxigenic *Escherichia coli*-infected piglet models, it was shown that the supplementation with GABA resulted in a notable increase in the growth of certain low-abundance microbes like *Enterococcus* and *Bacteroidetes*. It was demonstrated that GABA supplementation correlated with a substantial increase in the release of specific cytokines, including IL-4, IFN-γ, IL-1β, and IL-17, within the jejunal tissues of the piglets. These cytokines play a pivotal role in regulating Secretory Immunoglobulin A (SIgA), a critical antibody primarily present in mucosal surfaces and essential for maintaining intestinal mucosal immunity. Therefore, these findings suggest that GABA supplementation may enhance intestinal health by modulation of immune responses via cytokine regulation, thereby reducing gut inflammation [74]. Reduced inflammation can promote a more favorable environment for beneficial bacteria to thrive, as inflammation frequently contributes to an imbalance in gut microbiota, commonly referred to as dysbiosis [75].

**Figure 5:**
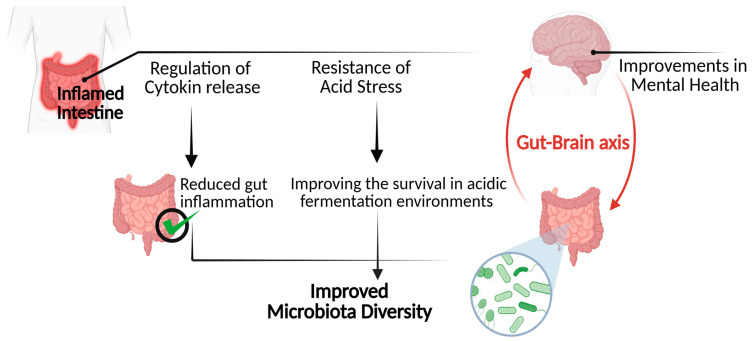
GABA’s mechanism of action in improving microbiota diversity.

As a matter of fact, beneficial bacteria inherently produce GABA as a metabolic byproduct of their activities within the gastrointestinal tract. The release of GABA enables these bacteria to resist acid stress, which is a critical factor for their colonization and survival in acidic fermentation environments within the gut [68]. Thereby highlighting the significance of GABA in enhancing and supporting the diversity of gut microbiota. Furthermore, improvements in mental health associated with neural GABAergic signaling may also exert a beneficial impact on gastrointestinal health. It is widely recognized that heightened levels of anxiety and stress are often associated with gut dysbiosis. This association is mediated by the modulation of the gut-brain axis, which refers to the bidirectional communication network connecting the gastrointestinal tract and the central nervous system. This connection emphasizes the significant role of GABA as a critical factor in gut microbiota diversity [68], Figure 5.

## 5. Sources and Potential Food Applications

GABA can be present in various foods (Table 2), particularly in cereals, legumes, pseudo-cereals, fruits, vegetables, tea, cocoa beans, and mushrooms. While certain varieties may exhibit elevated GABA levels, the overall concentrations are generally modest. For instance, cereals contain between 0.67 and 54.00 mg of GABA per 100 g, whereas legumes can range from 0.56 to 61.00 mg per 100 g. Notably, black soybeans have the highest GABA content recorded at 61.00 mg per 100 g, and fruits such as lychee may contain as much as 350.00 mg per 100 g. Tea demonstrates considerable variability in GABA concentration, with white tea exhibiting the highest levels, reaching up to 207.00 mg per 100 g. Additionally, mushrooms, including shiitake and oyster varieties, contribute significant amounts of GABA. Cocoa beans are also recognized for their high GABA content, which influences the GABA levels in chocolate products. In contrast, nuts typically exhibit low concentrations of GABA. Overall, the variation in GABA levels across food types can be attributed to several factors, including cultivar selection, climatic conditions, and agricultural practices [76].

Researchers are actively investigating methods to enhance the GABA content within food products. Improving the GABA content in foods can be effectively achieved through a variety of natural and technological methods. One key approach is germination, where soaking seeds in water initiates sprouting, significantly enhancing GABA levels by activating enzymes that convert glutamate to GABA. Another effective method is fermentation, which employs micro-organisms such as bacteria and yeast that possess the enzyme glutamic acid decarboxylase (GAD), facilitating the conversion of glutamate to GABA. Adjusting temperature and humidity conditions during food processing can also elevate GABA content by leveraging the stress response in plants, leading to increased synthesis of GABA.

Additionally, exposing plants to abiotic stress factors like salinity, drought, or temperature extremes can also trigger GABA production as a protective mechanism. The polyamine degradation pathway is another avenue where the breakdown of polyamines through enzymatic reactions results in GABA production. Furthermore, innovative technologies, including genetic engineering and advanced fermentation techniques, are being explored to enhance GABA content without compromising food quality. Consumers tend to prefer natural methods for GABA enrichment, as these approaches are perceived as safer and more acceptable, focusing on enhancing endogenous GABA levels rather than adding it externally, which is both economical and aligns with the growing consumer demand for natural foods [76,77,78]. These methods not only enhance the GABA content in food products but also align with consumer preferences for natural and health-promoting items. By employing these techniques, food manufacturers can develop GABA-enriched products that meet the increasing demand for functional foods that offer health benefits.

**Table 2:** GABA levels are present in various food categories, reflecting high and low concentrations.

Food Category	Food Item	GABA Content (mg/100 g)	Recommended Daily Intake *
Vegetables	Spinach	232.10–381.00	100–200 (mg day^−1^)
	Tomato	219.86–404.89	
	Sweet potato, cucumber, collard, and eggplant	<34.00 each	
Fruits	Lychee	170.00–350.00	
	Grape	58.93–109.83	
	Kiwifruit, Jujube fruit, and Mulberry fruit	<34.00 each	
Cocoa Beans		101.20	
Legumes	Black soybean	61.00	
	Lupin	46.00	
Pseudo-Cereals	Tartary buckwheat	42.60–112.5	
	Quinoa	7.00–66.10	
Cereals	Barley	54.00	
	Brown rice	27.00	
	Wheat and corn	≤15.50 each	
	Red rice, black rice, and millet	<10.00 each	
Edible Mushrooms	Oyster mushroom	32.15–57.73	
	Shiitake mushroom	17.00–35.00	
	White mushroom	18.00–20.00	
Tea	White tea	3.49–207.00	
	Oolong tea, green tea, and black tea	0.24–97.00 each	

Data was derived from Hou et al. [76]. *: A daily dosage is often suggested for benefits such as relaxation, improved sleep quality, and immune support [79,80,81]. It is imperative to consult a healthcare professional prior to initiating any supplementation to ensure safety and address individual health requirements.

## 6. Safety Considerations

GABA is well-known for its positive effects on relaxation, sleep quality, and anxiety reduction, along with its advantageous antidiabetic properties highlighted in the current review. However, excessive consumption of GABA may pose potential risks and side effects, as indicated by various research studies [82,83,84]. It was shown that elevated doses of GABA, particularly when taken in conjunction with substances like gamma-hydroxybutyric acid (GHB), may result in neurotoxicity. Research has demonstrated that prolonged and excessive use of GHB is associated with cognitive impairments, such as memory decline and reduced cognitive functions [82]. Another study showed that GABAergic compounds can exhibit paradoxical effects depending on the dosage. At low physiological concentrations, these compounds have been associated with anxiogenic effects, potentially inducing anxiety. Conversely, when administered at higher doses, they can exert anxiolytic effects, effectively reducing anxiety. This dual action underscores the critical importance of precise dosing to mitigate the risk of unintended psychological responses [83]. Moreover, gastrointestinal side effects have been observed as a consequence of high-dose oral GABA supplementation. A clinical trial examining the effects of natural GABA extracts indicated that several participants experienced adverse reactions, including abdominal discomfort and headaches [84]. These findings underscore the necessity for additional research to more comprehensively investigate the safety profile and tolerability of GABA supplementation at elevated dosages.

Furthermore, the response to GABAergic compounds can exhibit considerable variability among individuals. Research indicates that children and older adults may possess a heightened susceptibility to the adverse effects associated with excessive GABA intake. This increased vulnerability can be attributed to factors such as variations in metabolism, differences in body composition, and individual sensitivity to GABAergic compounds [82,83]. Additionally, the safety of GABA supplementation during pregnancy and lactation remains insufficiently addressed in the existing literature. Consequently, pregnant and breastfeeding individuals must exercise caution and seek informed guidance from their healthcare providers before considering the use of GABA supplements [85].

## 7. Future Insights

This review underscores the considerable potential of GABA as a viable agent for the management of metabolic disorders that affect a significant portion of the global population. However, it is essential to acknowledge that the majority of the research cited in this review is derived from animal studies, with only two human studies identified. Consequently, there is a pressing need for further investigations conducted in human populations to validate the beneficial effects observed in animal models thoroughly. It is essential to consider the individual variations in GABA responsiveness, which might be influenced by various factors. These include genetic differences that may affect GABA receptor function and signaling pathways, variations in diabetes subtypes that can alter metabolic responses or other personal health conditions. Future research should also consider additional critical factors to evaluate the efficacy of GABA in diabetes management. Specifically, the timing of administration, whether during fasting or postprandial periods, may influence the metabolic effects of GABA. To fully harness and optimize the therapeutic potential of GABA, future research must prioritize the development of meticulously designed dosage protocols that are tailored to accommodate the diverse needs of various patient populations. Furthermore, the combination therapy involving GABA and other substances requires additional in-depth investigation. On the other hand, the potential risks associated with overstimulating GABAergic pathways in conditions such as diabetes and metabolic disorders raise concerns regarding possible interference with different neurotransmitter systems.

## 8. Conclusions

GABA interventions in diabetic conditions are associated with significant improvements in glucose and insulin homeostasis, enhancements in lipid profiles, and reductions in organ dysfunctions related to diabetes. It was found that pancreatic GABAergic signaling can facilitate protective effects against β cell degradation by stimulating sustained transdifferentiation of α cells into β-like cells. Activated GABAergic signaling can further promote the activation of the PI3K/AKT signaling pathway, resulting in improved insulin signaling and maintained glucose homeostasis. Additionally, GABA can exert inhibitory effects on gene expressions related to gluconeogenesis and lipogenesis, contributing to enhanced glucose and lipid profiles. GABA may further enhance gut microbiota diversity by reducing gut inflammation through its anti-inflammatory and immunomodulatory properties, while also playing a role in modulating the gut-brain axis. Furthermore, as a primary neurotransmitter, GABA significantly aids in alleviating neural disorders associated with diabetes.

## Figures and Tables

**Table 1:** Antidiabetic effects of GABA interventions observed in diabetic individuals/models.

Study Design	Subjects Characteristics	GABA Treatment	Main Outcomes	Reference
Clinical trial (11-day duration)	Adult male individuals with long-standing T1D (*n* = 6; ave age 24.8 years; disease duration 14.7 years)	Oral administration of GABA tablet (Remygen) at escalating doses (200, 600, and 1200 mg) as a single daily dose while fasting, for three consecutive days.	- Significant hormonal response to hypoglycemia was noted with 600 mg of GABA, including increases in glucagon, epinephrine, growth hormone, and cortisol.	Espes et al. [23]
One-year randomized, double-blind, placebo-controlled trial	Children with newly diagnosed T1D; (*n* = 97; age 4–18 years)	Oral administration of GABA capsules or placebo at 1.5 g/day, divided into two daily doses (morning and evening) + injections of GAD or placebo (20 μg/dose) twice a day.	- GABA monotherapy did not maintain β-cell function, with no significant differences in glycemic control. - A combination of GABA with GAD showed a significant reduction in fasting and meal-stimulated serum glucagon levels after 12 months.	Martin et al. [24]
Animal trial	Streptozotocin-induced male Wistar rats (*n* = 12)	Oral administration of GABA tea extract at two doses, low and high (4.55 and 45.5 mg/kg BW), in 0.2 mL distilled water daily for six weeks.	- Significant reduction in fasting blood glucose levels. - Inhibition of cardiac fibrosis development.	Cherng et al. [25]
Animal trial	Streptozotocin-induced male Wistar rats (*n* = 9)	Intragastrical administration of GABA tea extract at two doses, low and high (3.01 and 30.1 µg/rat per day) for six weeks.	- Significant reduction in blood glucose levels (dose-dependently). - Reduction in cerebral cortex autophagy.	Huang et al. [26]
Animal trial	Streptozotocin-induced male C57BL/6 mice (*n* = 6)	Oral administration of GABA-rich yogurt fermented by *Streptococcus thermophilus* at three doses: (1, 2 and 4 g/L) for six weeks.	- Significant improvement in hyperglycemia (dose-dependently). - Enhancement in glucose tolerance. - Significant regulation of serum lipids. - Significant increase in insulin levels. - Normalization of liver and kidney hypertrophy. - Restoration of pancreatic islets.	Chen et al. [27]
Animal trial	HFD-fed + streptozotocin-induced male C57BL/6 mice (*n* = 8)	Oral administration of GABA-rich yogurt fermented by *Streptococcus thermophilus* at three doses: (0.5, 1, and 2 g/L) daily for 12 weeks.	- Significant improvements in insulin sensitivity (dose-dependently). - Enhancement in glucose tolerance. - Regulation of serum insulin and lipid levels. - Improvement in kidney function. - Restoration of pancreatic islets (dose-dependently).	Li et al. [28]
Animal trial	Streptozotocin-induced male Wistar rats (*n* = 10)	Oral administration of fermented *Hericium erinaceus* Juice (GABA dose: 1.27 g/kg) daily for 12 weeks (pre- and post-treatment).	- Significant improvement in plasma insulin levels. - Reduction in plasma glucose levels. - Improvements in glycated hemoglobin levels.	Chaiyasut et al. [29]
Animal trial	Streptozotocin-induced C57BL/6 mice (*n* = 8)	Intragastrical administration of two *Lactobacillus brevis* strains at 250 μL × 10^5^ CFU/mL/day (GABA conc. 0.05–1.98 g/L) for 4 weeks.	- Significant reduction in postprandial blood glucose levels. - Reduction in serum lipids. - Significant improvements in liver function.	Abdelazez et al. [30]
Animal trial	HFD-fed male Sprague–Dawley rats (*n* = 5)	15% of GABA-enriched wheat bran incorporated into the daily HFD for 8 weeks.	- Improvement in insulin resistance and glucose tolerance. - Alterations in key genes related to glucose metabolism.	Shang et al. [31]
Animal trial	HFD-fed + streptozotocin-induced male C57BL/6 mice (*n* = 8)	GABA-rich sprouted adzuki beans were incorporated into the daily HFD at 15, 25, and 35 g/100 g for 6 weeks.	- Significant reduction in fasting blood glucose. - Improvement in glycolipid metabolism. - Notable improvements in gut microbiota.	Zhang et al. [32]
Animal trial	HFD–induced hyperinsulinemia in male C57BL/6 mice (*n* = 5)	Oral administration of GABA at 6 mg/mL in drinking water for 4 weeks.	- Significant improvement in liver glucose metabolism. - Improvement in insulin resistance. - Promotion of pancreatic β-cells proliferation. - Prevention of lipid accumulation and promotion of fatty acid β oxidation in hepatocytes.	Chen et al. [33]
Animal trial	Streptozotocin-induced C57BL/6 mice (*n* = 6)	Intraperitoneal injection of GABA at 10 mg/kg per day for 10 days.	- Increase in pancreatic insulin stores. - Significant reduction in apoptotic β-cells percentage. - Enhancement in β-cell proliferation.	Sarnobat et al. [34]
Animal trial	Streptozotocin-induced wild-type mice (*n* = 6)	Intraperitoneal injection of GABA at 250 mg/kg daily for 6 months.	- Improved glucose tolerance. - Enhancements in β-cell proliferation.	Ben-Othman et al. [35]
Animal trial	HFD-fed + streptozotocin-induced male and female Wistar rats (*n* = 8)	Intraperitoneal injection of GABA at 1.5 g/kg per day during mating, pregnancy, and breastfeeding.	- Significant improvement in glucose tolerance and insulin resistance. - GABA treatment in dams indirectly reduced insulin resistance and improved glycemic regulation in their offspring compared to untreated diabetic dams.	Rezazadeh et al. [36]
Animal trial	HFD-fed + streptozotocin-induced male and female Wistar rats (*n* = 7)	Intraperitoneal injection of GABA at 1.5 g/kg per day + HFD feeding.	- Both dams and their offspring showed improvements in insulin tolerance, decreased glucose levels, reduced serum lipid concentrations, and enhanced liver glycogen levels.	Hosseini Dastgerdi et al. [37]
Animal trial	HFD-fed + streptozotocin-induced male Wistar rats (*n* = 10)	Intraperitoneal injection of GABA at 1.5 g/kg BW per day for three months.	- Significant improvement in glucose tolerance and insulin resistance. - Protective and regenerative effects on β-cells function.	Sohrabipour et al. [38]
Animal trial	NOD mice (*n* = 21–26)	Intragastrical administration of GABA at 1 mmol/kg (twice a day) and/or Rapamycin at 1 mg/kg (once a day) for 12 weeks.	- Combination therapy showed more protective effects on β-cells function compared to either monotherapy.	He et al. [39]
Experimental in vitro study	Ethanol-induced rat pancreatic β-cells (INS-1 cells)	Exposure to GABA at 100 μM for 2 h.	- Pretreating with GABA completely alleviated the inhibitory effect of ethanol on insulin release.	Wang et al. [40]

Abbreviations. GABA: γ-aminobutyric acid; T1D: type 1 diabetes; GAD: glutamic acid decarboxylase; ave: average; BW: body weight; C57BL/6: mouse strain utilized in immunology, cancer, and metabolic studies; HFD: high-fat diet; NOD mice: nonobese diabetic mouse model of autoimmune type 1 diabetes; INS-1 cells: insulin-secreting cell line.

## Data Availability

Not applicable.
